# Effects of phenytoin on ethanol-induced gastric ulceration in rats: involvement of oxidative stress, inflammation, and apoptosis

**DOI:** 10.1186/s40780-026-00582-2

**Published:** 2026-06-03

**Authors:** Kaveh Rahimi, Masoud Najafi, Shahrzad Fayazfard, Mehdi Esmaeili

**Affiliations:** Department of Basic Sciences, Faculty of Veterinary Medicine, Shahid Chamran University of Ahvaz, Ahvaz, Iran

**Keywords:** Phenytoin, Gastric ulcer, Ethanol, Oxidative stress, Inflammation, Apoptosis

## Abstract

**Background:**

Peptic ulcer disease is a common gastrointestinal disorder caused by an imbalance between damaging factors and mucosal protection. Alcohol induces gastric injury through oxidative stress, inflammation, and apoptosis, while phenytoin may have potential preventive effects in ulcer. This study aimed to evaluate the gastroprotective effects of phenytoin against ethanol-induced gastric ulcers in rats, focusing on oxidative stress, inflammatory, and apoptotic biomarkers, and to elucidate the underlying molecular mechanisms.

**Methods:**

Thirty-two male Wistar rats were randomly divided into four groups: control, ethanol-induced gastric ulcer (ETH), phenytoin-treated (ETH + Phen), and omeprazole-treated (ETH + Ome). Gastric ulcers were induced by oral administration of absolute ethanol. Ulcer area, gastric content volume, oxidative stress markers (TAC, MDA, GSH, SOD, CAT), inflammatory mediators (NF-κB, TNF-α, IL-1β, iNOS, COX-2), and apoptotic markers (Bax, Bcl-2, Caspase-3) were measured using biochemical assays, real time PCR, and ELISA.

**Results:**

Ethanol administration significantly increased ulcer area, gastric content volume, MDA levels, NF-κB activation, pro-inflammatory cytokines, and pro-apoptotic markers, while reducing TAC, GSH, and antioxidant enzyme activities. Preventive phenytoin treatment significantly attenuated these effects, reducing ulcer area, restoring antioxidant balance, suppressing NF-κB-mediated inflammation, and modulating apoptotic signaling. Both phenytoin and omeprazole improved these parameters compared with the ethanol group; however, no direct statistical comparison between the two treatments was performed.

**Conclusion:**

Phenytoin exhibits significant gastroprotective effects against ethanol-induced gastric injury, which may be associated with modulation of oxidative stress, inflammatory pathways, and apoptosis.

## Background

Peptic ulcer is one of the most prevalent gastrointestinal disorders in the modern era [1]. These ulcers are typically chronic, often solitary, and can develop in different regions of the gastrointestinal tract [2]. The pathogenesis of peptic ulcer disease (PUD) is multifactorial, primarily resulting from an imbalance between aggressive factors such as gastric acid and pepsin, and protective mechanisms of the mucosal lining, including adequate blood flow and prostaglandin production [3]. Several risk factors contribute to PUD, including psychological stress, alcohol consumption, cigarette smoking, Helicobacter pylori infection, and the use of nonsteroidal anti-inflammatory drugs (NSAIDs) [4].

In 2004, alcohol was responsible for nearly 4% of all deaths worldwide, which corresponded to about 2.25 million fatalities. Regarding the gastrointestinal tract, alcohol-related mortality accounted for 7.0% of esophageal cancers, 3.4% of mouth and oropharynx cancers, and 0.8% of colon and rectal cancers during the same year [5]. Clinically, alcohol intake can cause heartburn, nausea, and gastric discomfort, largely due to reflux of corrosive gastric substances, reactive oxygen species (ROS) production, and lysosomal enzyme release [6, 7]. These observations suggest that alcohol induces oxidative stress, a critical factor in alcohol-related diseases [8]. Indeed, cells exposed to ethanol produce ROS [9], and oxidative stress is also linked to esophageal disorders [10]. In addition to oxidative stress, ethanol-induced gastric damage is closely associated with activation of inflammatory signaling pathways. Ethanol has been shown to stimulate nuclear factor kappa B (NF-κB), a key transcription factor regulating the expression of pro-inflammatory mediators such as tumor necrosis factor-alpha (TNF-α), interleukin-1 beta (IL-1β), inducible nitric oxide synthase (iNOS), and cyclooxygenase-2 (COX-2) [11–14]. Overactivation of these mediators exacerbates mucosal inflammation, increases nitrosative stress, and further amplifies tissue injury. Moreover, accumulating evidence indicates that ethanol-induced oxidative and inflammatory insults ultimately trigger apoptotic pathways in gastric epithelial cells, as reflected by increased expression of pro-apoptotic proteins such as Bax and caspase-3 and suppression of anti-apoptotic proteins like Bcl-2 [14].

Moreover, bile acids and/or gastric acids are known to provoke oxidative stress and disrupt signaling pathways including mitogen-activated protein kinase (MAPK), nuclear factor-kappa B (NF-κB), and Signal Transducer and Activator of Transcription 3 (STAT3).

Phenytoin received FDA approval in 1939 for managing epilepsy. Although it possesses a narrow therapeutic window, it remains widely utilized for controlling generalized tonic-clonic seizures, complex partial seizures, status epilepticus, trigeminal neuralgia, and certain behavioral conditions. Historically, phenytoin was also employed as an anti-arrhythmic agent and for treating toxicity caused by digoxin and tricyclic antidepressants; however, its use in these areas has become outdated [15]. Importantly, phenytoin has demonstrated therapeutic potential in the treatment of ulcers [16, 17].

Phenytoin has been reported to enhance tissue repair, reduce inflammation, and promote wound healing in various experimental and clinical contexts [18–21]. These properties suggest that it may have potential therapeutic relevance in gastric mucosal injury; however, its effects and underlying mechanisms in ethanol-induced gastric ulceration remain insufficiently explored. This study aimed to evaluate the gastroprotective effects of phenytoin against ethanol-induced gastric ulceration in rats, with particular emphasis on oxidative stress, inflammatory, and apoptotic biomarkers. Furthermore, the study sought to elucidate the underlying molecular mechanisms through which phenytoin preserves gastric mucosal integrity and mitigates ethanol-induced gastric injury.

## Methods

### Animals

In this study, 32 male Wistar rats (220–250 g) were used. The animals were housed in groups under standard laboratory conditions, including a temperature of 22 ± 2 °C, a 12-hour light–dark cycle, and free access to food and water. All experimental procedures were conducted in accordance with the ARRIVE (Animal Research: Reporting In Vivo Experiments) guidelines [22]. Efforts were made to minimize animal suffering and reduce the number of animals used in accordance with the 3Rs principle (Replacement, Reduction, and Refinement).

### Experimental design

The rats were randomly assigned to four groups (*n* = 8). The groups were organized as follows: (1) Control Group: Rats in this group were injected intraperitoneally (i.p.) with distilled water for 14 days. On day 14, they were given oral saline. (2) Ethanol-induced gastric ulcer (ETH) group: These rats were also injected with distilled water i.p. for 14 days, but on day 14, they were administered ethanol orally to induce gastric ulcers. (3) Phenytoin-treated (ETH + Phen) group: Rats in this group received an i.p. injection of phenytoin (Caspian Pharmaceutical Company, Iran) at a dosage of 50 mg/kg body weight [23] for 14 days. On day 14, they were given oral ethanol to induce gastric ulcers. (4) Omeprazole-treated (ETH + Ome) group: Rats were injected i.p. with omeprazole at a dosage of 10 mg/kg body weight for 14 days. On day 14, they received oral ethanol to induce gastric ulcers.

### Induction of gastric ulcer

Gastric ulcers were induced by oral administration of absolute ethanol (96%, Merck, Germany). Each rat received 1 mL per 200 g body weight via orogastric gavage. Animals were fasted for 24 h with free access to water prior to ethanol administration to ensure an empty stomach and enhance lesion formation [24, 25].

### Tissue collection

After the final ethanol administration, rats were anesthetized with xylazine (10 mg/kg) and ketamine (80 mg/kg, i.p.), euthanized, and the stomachs were excised, washed with ice-cold saline, and dissected longitudinally. Tissue samples for biochemical assays, RNA extraction, and protein quantification were immediately snap-frozen in liquid nitrogen and stored at − 80 °C until analysis.

### Measurement of ulcer area

To assess ulcer area, the stomachs were opened longitudinally, and the lesions were measured in millimeters based on the total stomach surface [26].

### Measurement of gastric content volume

The stomach contents were extracted using 5 mL of saline, which was collected in a container. The volume of gastric contents was then measured to evaluate changes related to tissue damage and acid production in the stomach.

### Antioxidant enzyme activity

Total Antioxidant Capacity (TAC) indicates the ability of gastric tissue to neutralize free radicals and protect cells from oxidative damage. TAC was measured using a colorimetric assay per manufacturer’s instructions. Results were expressed as µmol Trolox equivalents/g tissue. Malondialdehyde (MDA), an index of lipid peroxidation, was measured using a colorimetric kit (ZellBio GmbH, Germany), expressed as nmol/g tissue. Reduced glutathione (GSH) was quantified using a commercial kit (ZellBio GmbH, Germany), expressed as µmol/g tissue. Superoxide dismutase (SOD) activity was assessed with a ZellBio kit, expressed as U/mg protein, where one unit represents enzyme activity inhibiting the reaction by 50%. Catalase (CAT) activity was measured using a colorimetric kit, expressed as U/mg protein. All measurements were performed according to the manufacturer’s instructions, using a microplate reader.

### Real-time PCR

Total RNA was extracted using an RNA kit (Pars Tous, Iran). RNA concentration and purity were assessed using a NanoDrop spectrophotometer (260/280 nm). cDNA was synthesized from 1 µg RNA, and quantitative real-time PCR (RT-qPCR) was performed using SYBR Green-based master mix (Pars Tous, Iran) in a 20 µL reaction volume. mRNA levels of NF-κB, iNOS, COX-2, Bax, Bcl-2, and Caspase-3 were quantified. GAPDH was used as the internal reference gene. Relative expression was calculated using the 2^−ΔΔCt method. Primer sequences are listed in Table 1. Reactions were performed in duplicate with negative controls.

**Table 1:** The primer sequences

Primer name	Sequence (5′ → 3′)
GAPDH-rat-F	AGTTCAACGGCACAGTCAAG
GAPDH-rat-R	TACTCAGCACCAGCATCACC
NFKB-rat-F	TCAACATGGCAGACGACGAT
NFKB-rat-R	TTGAAGGTATGGGCCATCTGT
iNOS-rat-F	CACGACACCC TTCACCACAAG
iNOS-rat-R	TTGAGGCAGAAGCTCCTCCA
cox2-rat-F	TGAGCAACTATTCCCAAACC
cox2-rat-R	CATTGTGTTGGTGGTTTCGG

### ELISA

Pro-inflammatory cytokines IL-1β and TNF-α were quantified in gastric tissue using commercial ELISA kits (Red Biotechnology, Kiazist, Hamedan, Iran). Gastric tissues were homogenized in cold PBS (pH 7.4) and centrifuged at 10,000 × g for 10 min at 4 °C. Protein concentration was measured by Bradford assay (Bio-Rad, USA), and cytokine levels were normalized to total protein (pg/mg protein). Supernatants were added to antibody-coated microplates, incubated, washed, and developed with substrate solution. Absorbance was read at 450 nm, and cytokine concentrations were calculated from standard curves. All assays were performed in duplicate according to the manufacturer’s instructions.

### Statistical analysis

Data were analyzed using GraphPad Prism 8. Normality was checked with the Shapiro–Wilk test. One-way ANOVA was used to compare means, followed by Tukey’s post-hoc test for pairwise comparisons. A p-value < 0.05 was considered statistically significant. Data are presented as mean ± SEM.

## Results

### Ulcer area

**Fig. 1:**
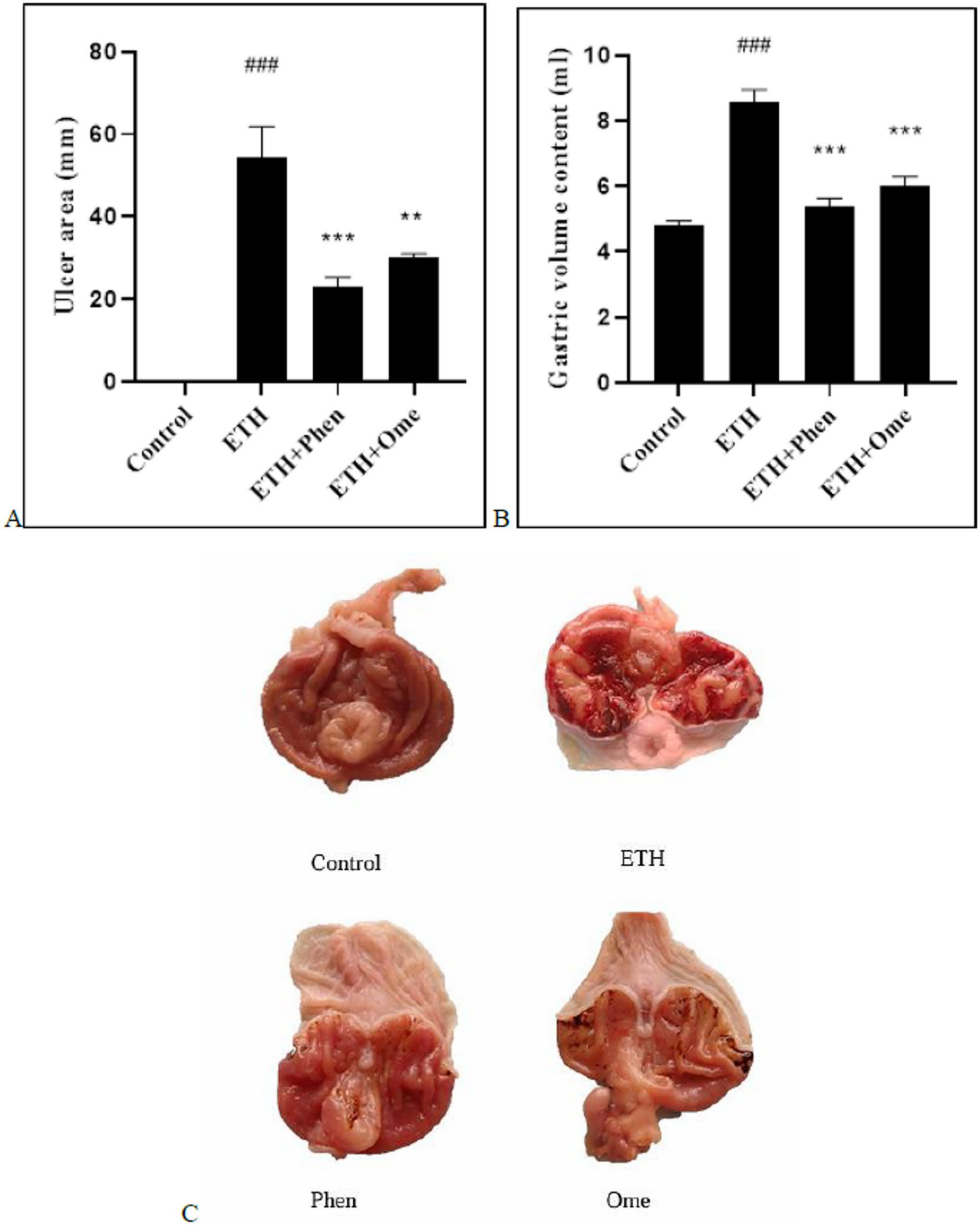
Effects of phenytoin on (**A**) ulcer area, (**B**) gastric content volume, and (**C**) macroscopic appearance of stomachs. Data are presented as mean ± SEM (*n* = 8). Experimental groups included control, ethanol (ETH), phenytoin (50 mg/kg) + ETH, and omeprazole (10 mg/kg) + ETH. #*p* < 0.05, ##*p* < 0.01, and ###*p* < 0.001 indicate significant differences compared with the control group. *p < 0.05, **p < 0.01, and ***p < 0.001 indicate significant differences compared with the ETH group

The ulcer area differed significantly among the experimental groups (one-way ANOVA, *F*(3,28) = 34.15, *p* < 0.0001, *R²* = 0.8367). The ETH group exhibited a markedly larger ulcer area (54.50 ± 7.31) compared with the control group (0.00 ± 0.00; *p* < 0.001). Co-treatment with phenytoin significantly reduced the ulcer area in the ETH + Phen group (23.00 ± 2.24) relative to the ETH group (*p* < 0.01). Similarly, omeprazole administration significantly decreased the ulcer area in the ETH + Ome group (30.17 ± 0.79) compared with the ETH group (*p* < 0.05) (Fig. 1A and D).

### Gastric volume content

The volume of gastric contents showed significant differences among the experimental groups (one-way ANOVA, *F*(3,28) = 38.08, *p* < 0.0001, *R²* = 0.8510). The ETH group exhibited a significantly higher gastric content volume (8.58 ± 0.37) compared with the control group (4.81 ± 0.13; *p* < 0.001). In contrast, co-administration of phenytoin markedly reduced the gastric content volume in the ETH + Phen group (5.40 ± 0.23) relative to the ETH group (*p* < 0.001). Similarly, treatment with omeprazole significantly decreased the gastric content volume in the ETH + Ome group (6.02 ± 0.28) compared with the ETH group (*p* < 0.001) (Fig. 1C).

### Oxidative stress

**Fig. 2:**
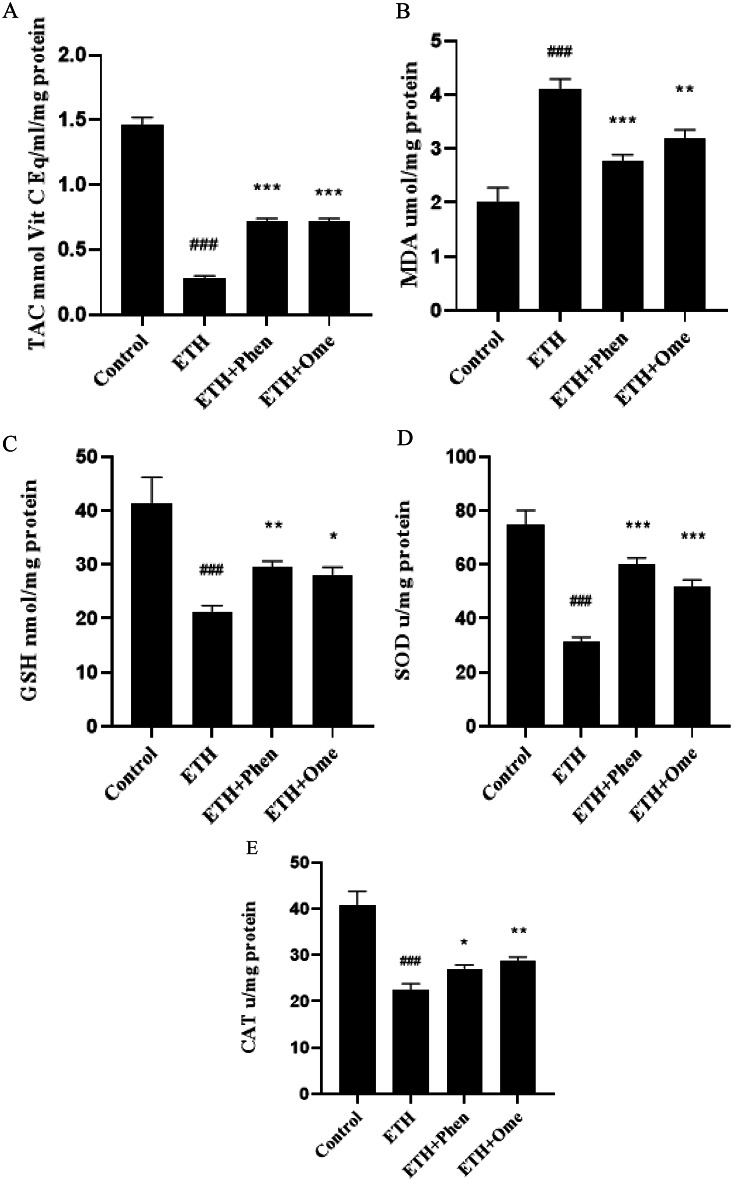
Effects of phenytoin on oxidative stress parameters: (**A**) TAC, (**B**) MDA, (**C**) GSH, (**D**) SOD, and (**E**) CAT levels. Data are presented as mean ± SEM (*n* = 8). Experimental groups included control, ethanol (ETH), phenytoin (50 mg/kg) + ETH, and omeprazole (10 mg/kg) + ETH. *p* < 0.05, ##*p* < 0.01, and ###*p* < 0.001 indicate significant differences compared with the control group. *p < 0.05, **p < 0.01, and ***p < 0.001 indicate significant differences compared with the ETH group

TAC levels differed significantly among the experimental groups (one-way ANOVA, *F*(3,28) = 200.2, *p* < 0.0001, *R²* = 0.9678). The ETH group exhibited a pronounced reduction in TAC levels (0.28 ± 0.02) compared with the control group (1.46 ± 0.06; *p* < 0.001). In contrast, phenytoin significantly increased TAC levels in the ETH + Phen group (0.72 ± 0.02) relative to the ETH group (*p* < 0.001). Similarly, administration of omeprazole significantly elevated TAC levels in the ETH + Ome group (0.72 ± 0.02) compared with the ETH group (*p* < 0.001) (Fig. 2A).

MDA levels differed significantly among the experimental groups (one-way ANOVA, *F*(3,28) = 36.41, *p* < 0.0001, *R²* = 0.8452). The ETH group showed a significant increase in MDA levels (4.11 ± 0.19) compared with the control group (2.01 ± 0.11; *p* < 0.001). Phenytoin significantly reduced MDA levels in the ETH + Phen group (2.77 ± 0.12) relative to the ETH group (*p* < 0.001). Similarly, administration of omeprazole significantly decreased MDA levels in the ETH + Ome group (3.20 ± 0.15) compared with the ETH group (*p* < 0.001) (Fig. 2B).

GSH levels showed significant differences among the experimental groups (one-way ANOVA, *F*(3,28) = 32.92, *p* < 0.0001, *R²* = 0.8316). The ETH group exhibited a marked reduction in GSH levels (21.17 ± 1.23) compared with the control group (41.35 ± 1.98; *p* < 0.001). Phenytoin significantly increased GSH levels in the ETH + Phen group (29.59 ± 1.07) relative to the ETH group (*p* < 0.001). Similarly, administration of omeprazole significantly elevated GSH levels in the ETH + Ome group (28.12 ± 1.40) compared with the ETH group (*p* < 0.001) (Fig. 2C).

SOD activity differed significantly among the experimental groups (one-way ANOVA, *F*(3,28) = 67.47, *p* < 0.0001, *R²* = 0.9101). The ETH group exhibited a pronounced reduction in SOD activity (31.39 ± 1.61) compared with the control group (74.90 ± 2.14; *p* < 0.001). Phenytoin significantly restored SOD activity in the ETH + Phen group (60.29 ± 2.14) relative to the ETH group (*p* < 0.001). Similarly, administration of omeprazole significantly increased SOD activity in the ETH + Ome group (51.52 ± 2.80) compared with the ETH group (*p* < 0.001) (Fig. 2D).

CAT activity differed significantly among the experimental groups (one-way ANOVA, *F*(3,28) = 54.75, *p* < 0.0001, *R²* = 0.8914). The ETH group showed a significant decrease in CAT activity (22.49 ± 1.32) compared with the control group (40.75 ± 1.23; *p* < 0.001). Phenytoin significantly increased CAT activity in the ETH + Phen group (27.06 ± 0.79) relative to the ETH group (*p* < 0.001). Similarly, administration of omeprazole significantly elevated CAT activity in the ETH + Ome group (28.86 ± 0.75) compared with the ETH group (*p* < 0.001) (Fig. 2E).

### NF-kB mRNA expressions

**Fig. 3:**
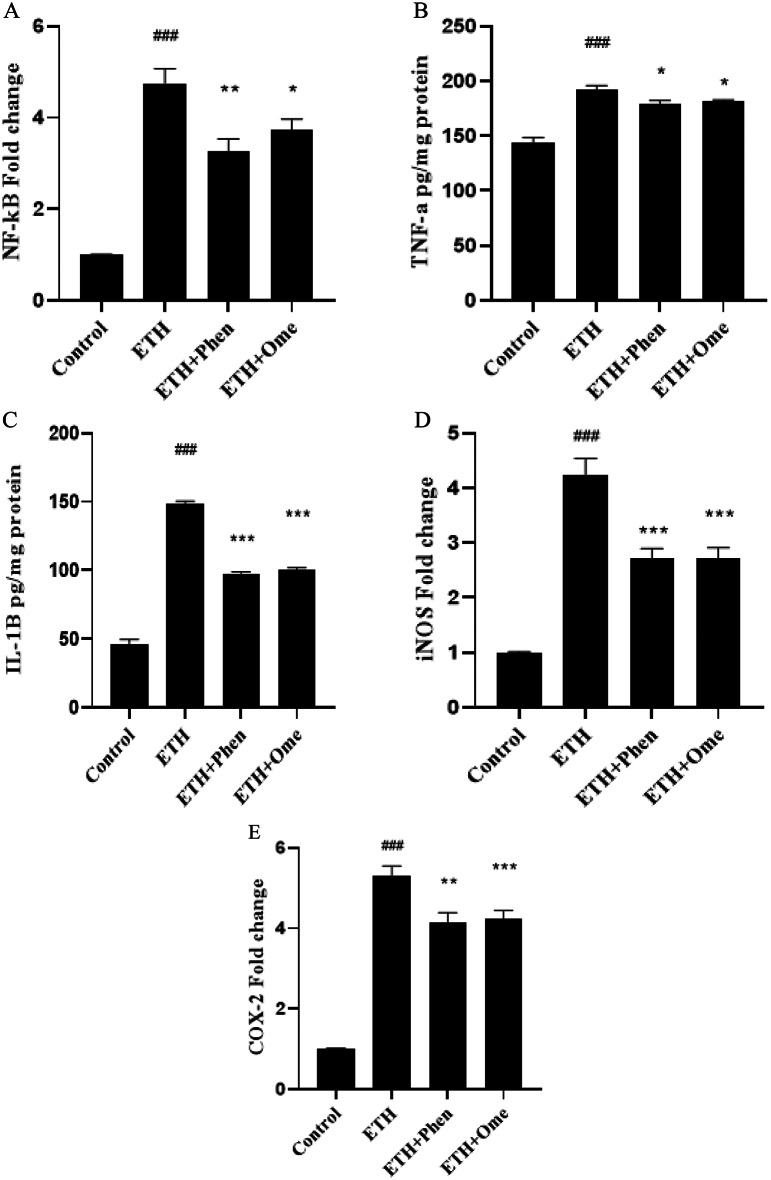
Effects of phenytoin on inflammatory marker expression: (**A**) NF-κB, (**B**) TNF-α, (**C**) IL-1β, (**D**) iNOS, and (**E**) COX-2. Data are presented as mean ± SEM (*n* = 8). Experimental groups included control, ethanol (ETH), phenytoin (50 mg/kg) + ETH, and omeprazole (10 mg/kg) + ETH. #*p* < 0.05, ##*p* < 0.01, and ###*p* < 0.001 indicate significant differences compared with the control group. *p < 0.05, **p < 0.01, and ***p < 0.001 indicate significant differences compared with the ETH group

NF-κB mRNA expressions differed significantly among the experimental groups (one-way ANOVA, *F*(3,28) = 43.91, *p* < 0.0001, *R²* = 0.8682). The ETH group exhibited a marked increase in NF-κB mRNA expressions (4.75 ± 0.32) compared with the control group (1.00 ± 0.00; *p* < 0.001). Phenytoin significantly reduced NF-κB levels in the ETH + Phen group (3.27 ± 0.26) relative to the ETH group (*p* < 0.001). Similarly, administration of omeprazole significantly decreased NF-κB mRNA expressions in the ETH + Ome group (3.73 ± 0.24) compared with the ETH group (*p* < 0.001) (Fig. 3A).

### TNF-α, IL-1B iNOS, and COX-2 levels

TNF-α levels differed significantly among the experimental groups (one-way ANOVA, *F*(3,28) = 56.59, *p* < 0.0001, *R²* = 0.8946). The ETH group exhibited a significant elevation in TNF-α levels (192.6 ± 3.54) compared with the control group (143.2 ± 2.13; *p* < 0.001). Phenytoin significantly reduced TNF-α levels in the ETH + Phen group (178.8 ± 3.52) relative to the ETH group (*p* < 0.001). Similarly, administration of omeprazole significantly decreased TNF-α levels in the ETH + Ome group (181.3 ± 1.64) compared with the ETH group (*p* < 0.001) (Fig. 3B).

IL-1β levels differed significantly among the experimental groups (one-way ANOVA, *F*(3,28) = 750.4, *p* < 0.0001, *R²* = 0.9912). The ETH group showed a pronounced increase in IL-1β levels (148.4 ± 2.14) compared with the control group (45.52 ± 1.60; *p* < 0.001). Phenytoin significantly reduced IL-1β levels in the ETH + Phen group (97.82 ± 1.19) relative to the ETH group (*p* < 0.001). Similarly, administration of omeprazole significantly decreased IL-1β levels in the ETH + Ome group (101.0 ± 0.92) compared with the ETH group (*p* < 0.001) (Fig. 3C).

iNOS levels differed significantly among the experimental groups (one-way ANOVA, *F*(3,28) = 43.67, *p* < 0.0001, *R²* = 0.8675). The ETH group exhibited a marked increase in iNOS levels (4.23 ± 0.31) compared with the control group (0.995 ± 0.008; *p* < 0.001). Phenytoin significantly reduced iNOS levels in the ETH + Phen group (2.72 ± 0.17) relative to the ETH group (*p* < 0.001). Similarly, administration of omeprazole significantly decreased iNOS levels in the ETH + Ome group (2.72 ± 0.19) compared with the ETH group (*p* < 0.001) (Fig. 3D).

COX-2 levels differed significantly among the experimental groups (one-way ANOVA, *F*(3,28) = 82.97, *p* < 0.0001, *R²* = 0.9256). The ETH group exhibited a pronounced increase in COX-2 levels (5.29 ± 0.26) compared with the control group (1.00 ± 0.006; *p* < 0.001). Phenytoin significantly reduced COX-2 levels in the ETH + Phen group (4.15 ± 0.24) relative to the ETH group (*p* < 0.001). Similarly, administration of omeprazole significantly decreased COX-2 levels in the ETH + Ome group (4.25 ± 0.20) compared with the ETH group (*p* < 0.001) (Fig. 3E).

### Bax, Bcl-2 and Caspase 3 expressions

**Fig. 4:**
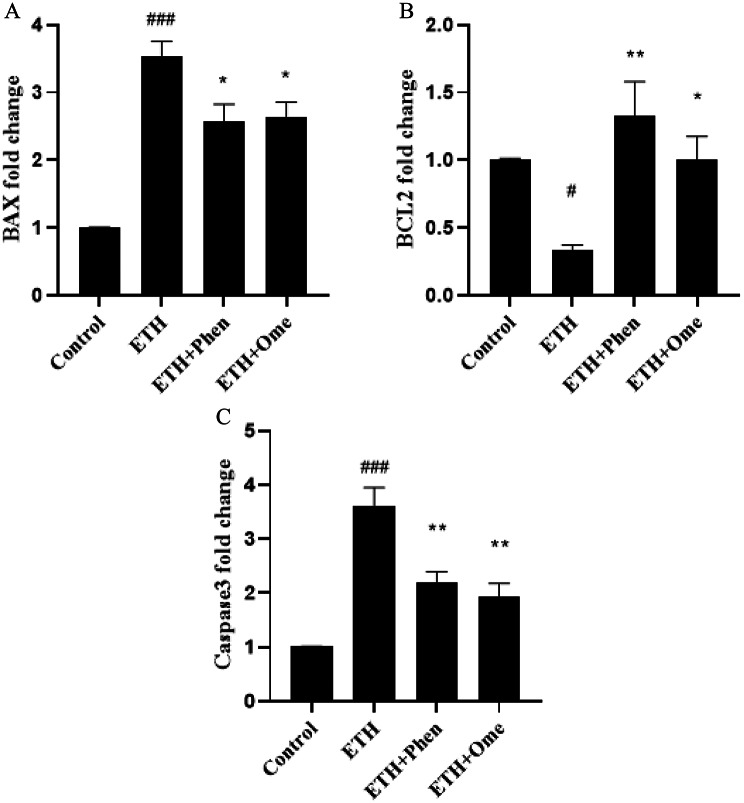
Effects of phenytoin on apoptotic markers: (**A**) Bax, (**B**) Bcl-2, and (**C**) Caspase-3 expression. Data are presented as mean ± SEM (*n* = 8). Experimental groups included control, ethanol (ETH), phenytoin (50 mg/kg) + ETH, and omeprazole (10 mg/kg) + ETH. #*p* < 0.05, ##*p* < 0.01, and ###*p* < 0.001 indicate significant differences compared with the control group. *p < 0.05, **p < 0.01, and ***p < 0.001 indicate significant differences compared with the ETH group

Bax expression differed significantly among the experimental groups (one-way ANOVA, *F*(3,28) = 27.81, *p* < 0.0001, *R²* = 0.8742). The ETH group exhibited a marked increase in Bax levels (3.54 ± 0.22) compared with the control group (1.00 ± 0.009; *p* < 0.001). Phenytoin significantly reduced Bax expression in the ETH + Phen group (2.58 ± 0.25) relative to the ETH group (*p* < 0.001). Similarly, administration of omeprazole significantly decreased Bax levels in the ETH + Ome group (2.65 ± 0.22) compared with the ETH group (*p* < 0.001) (Fig. 4A).

Bcl-2 expression differed significantly among the experimental groups (one-way ANOVA, *F*(3,28) = 7.23, *p* = 0.005, *R²* = 0.644). The ETH group exhibited a marked reduction in Bcl-2 levels (0.34 ± 0.03) compared with the control group (1.01 ± 0.006; *p* < 0.01). Phenytoin significantly restored Bcl-2 expression in the ETH + Phen group (1.33 ± 0.26) relative to the ETH group (*p* < 0.05). Similarly, administration of omeprazole normalized Bcl-2 levels in the ETH + Ome group (1.01 ± 0.17) compared with the ETH group (*p* < 0.05) (Fig. 4B).

Caspase-3 expression differed significantly among the experimental groups (one-way ANOVA, *F*(3,28) = 43.67, *p* < 0.0001, *R²* = 0.868). The ETH group exhibited a marked increase in Caspase-3 levels (3.60 ± 0.35) compared with the control group (1.01 ± 0.009; *p* < 0.001). Phenytoin significantly reduced Caspase-3 expression in the ETH + Phen group (2.21 ± 0.19) relative to the ETH group (*p* < 0.001). Similarly, administration of omeprazole significantly decreased Caspase-3 levels in the ETH + Ome group (1.94 ± 0.23) compared with the ETH group (*p* < 0.001) (Fig. 4C).

## Discussion

The findings of the present study suggest that ethanol administration may induce gastric mucosal injury, as reflected by increases in ulcer area, gastric content volume, oxidative stress, inflammatory mediators, and apoptotic signaling. The observed elevation in ulcer area in the ETH group, together with a reduction in total antioxidant capacity (TAC) and antioxidant enzyme activities (SOD and CAT), along with an increase in lipid peroxidation markers such as MDA, could indicate a role of oxidative stress in ethanol-induced gastric ulceration. Furthermore, ethanol-related upregulation of NF-κB and pro-inflammatory mediators, including TNF-α, IL-1β, iNOS, and COX-2, may reflect activation of inflammatory pathways contributing to mucosal injury. The increased expression of pro-apoptotic proteins (Bax, Caspase-3) and the decreased levels of the anti-apoptotic protein Bcl-2 suggest a potential involvement of apoptosis in ethanol-mediated gastric mucosal damage.

Co-treatment with phenytoin or omeprazole appeared to attenuate these pathological changes, indicating possible gastroprotective effects through modulation of oxidative stress, inflammatory responses, and apoptotic pathways. However, these observations are primarily associative, and the data do not establish definitive causality.

Ethanol-induced gastric injury is consistently linked to oxidative stress, inflammation, and apoptosis, as confirmed in both our findings and previous studies. Ethanol exposure increases ROS production, depletes GSH, and elevates lipid peroxidation products such as MDA and 4-hydroxynonenal (HNE), thereby disrupting cellular homeostasis and impairing tissue repair [14]. Additionally, ethanol activates NF-κB signaling, upregulating pro-inflammatory cytokines including TNF-α, IL-1β, IL-6, COX-2, and iNOS, which amplifies inflammatory responses and promotes apoptotic signaling via Bax and Caspase-3, while downregulating Bcl-2 [11–14].

The oxidative stress induced by ethanol metabolism involves both microsomal and mitochondrial pathways, generating ROS and reactive nitrogen species (RNS) that create a pro-oxidant cellular environment. This process results in intracellular GSH depletion and a reduction in overall antioxidant capacity. Concurrently, lipid peroxidation products interact with proteins, lipids, and nucleic acids, further contributing to cellular injury [27].

ROS-mediated activation of NF-κB signaling plays a key role in inflammatory gene regulation, including TNF-α, IL-1β, iNOS, and COX-2 expression [26–36]. Both the canonical and non-canonical NF-κB pathways contribute to this process, with IκB degradation allowing NF-κB translocation into the nucleus and transcriptional regulation of target genes, while the non-canonical pathway involves NF-κB2 (p100)/RelB complexes activated by specific receptors such as BAFFR, CD40, and RANK [37–39]. Our findings demonstrated that ethanol exposure significantly increased NF-κB mRNA expression and upregulated its downstream pro-inflammatory mediators, contributing to apoptosis and delayed tissue repair.

Phenytoin has been reported to enhance tissue repair, reduce inflammation, and promote wound healing in various experimental and clinical contexts [18–21]. In the present study, phenytoin treatment effectively reduced ulcer area, restored antioxidant balance (TAC, SOD, CAT), suppressed NF-κB activation, lowered pro-inflammatory cytokine levels (IL-1β, TNF-α, iNOS, COX-2), and modulated apoptotic signaling (decreased Bax and Caspase-3, restored Bcl-2). These findings suggest that phenytoin not only modulates oxidative stress and inflammation but also directly protects gastric epithelial cells from apoptosis.

Although phenytoin showed protective effects broadly similar to omeprazole, it is important to note that no direct statistical comparison between the two treatments was performed; therefore, this similarity should be interpreted with caution. Moreover, since phenytoin was administered for 14 days prior to ethanol exposure, the study primarily evaluates preventive rather than therapeutic effects, and further studies are warranted to assess its potential as a treatment for established gastric ulcers.

This study has several limitations. Assessment of gastric mucosal injury relied mainly on macroscopic and biochemical parameters, without detailed histopathological evaluation, which may limit the comprehensive characterization of tissue damage and repair. Additionally, the mechanisms by which phenytoin exerts its gastroprotective effects require further mechanistic investigations, including protein-level signaling pathway analyses.

## Conclusion

In conclusion, ethanol induces oxidative damage, inflammation, and apoptosis in the gastric mucosa, impairing tissue repair. Phenytoin, similarly to omeprazole, provides significant gastroprotective effects, including reduction of ulcer area, improvement of mucosal repair, suppression of NF-κB activation, and downregulation of pro-inflammatory cytokines. These results support the potential of phenytoin as a preventive therapeutic agent for ethanol-induced gastric injury, although further studies are needed to confirm its efficacy and clarify the underlying molecular mechanisms.

## Data Availability

Data will be made available on request.

## References

[1] Mota K, Dias G, Pinto M, Ferreira A, Brito A, Lima Filho C: J., Batista L. Flavonoids With Gastroprotective Activity. Molecules. 2009;20:979–1012. 10.3390/molecules14030979 PMC6253827 19305355

[2] Kumar V, Cotran RS. Robbins’ basic pathology. Arch Pathol Lab Med. 1994;118(2):203–203.

[3] Vonkeman HE, Klok RM, Postma MJ, Brouwers JR, Van De Laar MA. Direct medical costs of serious gastrointestinal ulcers among users of NSAIDs. Drugs Aging. 2007;24(8):681–90. 17702536 10.2165/00002512-200724080-00005

[4] Bafna P, Balaraman R. Anti-ulcer and antioxidant activity of DHC-1, a herbal formulation. J Ethnopharmacol. 2004;90(1):123–7. 14698519 10.1016/j.jep.2003.09.036

[5] Health WHODM, Abuse S. Global status report on alcohol 2004. World Health Organization; 2004.

[6] Li Y, Wo JM, Ellis S, Ray MB, Jones W, Martin RC. A novel external esophageal perfusion model for reflux esophageal injury. Dig Dis Sci. 2006;51(3):527–32. 16614962 10.1007/s10620-006-3165-4

[7] Oh T-Y, Lee J-S, Ahn B-O, Cho H, Kim W-B, Kim Y-B, Surh Y-J, Cho S-W. Hahm K-B: Oxidative damages are critical in pathogenesis of reflux esophagitis: implication of antioxidants in its treatment. Free Radic Biol Med. 2001;30(8):905–15. 11295533 10.1016/s0891-5849(01)00472-5

[8] Dey A, Cederbaum AI. Alcohol and oxidative liver injury. Hepatology. 2006;43(S1):S63–74. 16447273 10.1002/hep.20957

[9] Matharu Z, Enomoto J, Revzin A. Miniature enzyme-based electrodes for detection of hydrogen peroxide release from alcohol-injured hepatocytes. Anal Chem. 2013;85(2):932–9. 23163580 10.1021/ac3025619

[10] Goldman A, Chen HDR, Roesly HB, Hill KA, Tome ME, Dvorak B, Bernstein H, Dvorak K. Characterization of squamous esophageal cells resistant to bile acids at acidic pH: implication for Barrett’s esophagus pathogenesis. Am J Physiology-Gastrointestinal Liver Physiol. 2011;300(2):G292–302. 10.1152/ajpgi.00461.2010 PMC3043651 21127259

[11] Dvorak K, Chavarria M, Payne CM, Ramsey L, Crowley-Weber C, Dvorakova B, Dvorak B, Bernstein H, Holubec H, Sampliner RE. Activation of the interleukin-6/STAT3 antiapoptotic pathway in esophageal cells by bile acids and low pH: relevance to barrett’s esophagus. Clin Cancer Res. 2007;13(18):5305–13. 17875759 10.1158/1078-0432.CCR-07-0483

[12] Souza RF, Shewmake K, Terada LS, Spechler SJ. Acid exposure activates the mitogen-activated protein kinase pathways in Barrett’s esophagus. Gastroenterology. 2002;122(2):299–307. 11832445 10.1053/gast.2002.30993

[13] Abdel-Latif MM, O’Riordan J, Windle HJ, Carton E, Ravi N, Kelleher D, Reynolds JV. NF-κB activation in esophageal adenocarcinoma: relationship to Barrett’s metaplasia, survival, and response to neoadjuvant chemoradiotherapy. Ann Surg. 2004;239(4):491–500. 15024310 10.1097/01.sla.0000118751.95179.c6 PMC1356254

[14] Ren S, Wei Y, Wang R, Wei S, Wen J, Yang T, Chen X, Wu S, Jing M, Li H, et al. Rutaecarpine ameliorates ethanol-induced gastric mucosal injury in mice by modulating genes related to inflammation, oxidative stress and apoptosis. Front Pharmacol. 2020;11–2020. 10.3389/fphar.2020.600295 PMC7726440 33324227

[15] Craig S. Phenytoin poisoning. Neurocrit Care. 2005;3(2):161–70. 16174888 10.1385/NCC:3:2:161

[16] Hollisaz MT, Khedmat H, Yari F. A randomized clinical trial comparing hydrocolloid, phenytoin and simple dressings for the treatment of pressure ulcers [ISRCTN33429693]. BMC Dermatol. 2004;4(1):18. 15601464 10.1186/1471-5945-4-18 PMC545970

[17] Hokkam E, El-Labban G, Shams M, Rifaat S, El-mezaien M. The use of topical phenytoin for healing of chronic venous ulcerations. Int J Surg. 2011;9(4):335–8. 21338720 10.1016/j.ijsu.2011.02.007

[18] Bhatia A, Prakash S. Topical phenytoin for wound healing. Dermatol Online J. 2004;10(1):5. 15347487

[19] Pendse AK, Sharma A, Sodani A, Hada S. Topical phenytoin in wound healing. Int J Dermatol. 1993;32(3):214–7. 8444538 10.1111/j.1365-4362.1993.tb02799.x

[20] Shaw J, Hughes CM, Lagan KM, Bell PM. The clinical effect of topical phenytoin on wound healing: a systematic review. Br J Dermatol. 2007;157(5):997–1004. 17854378 10.1111/j.1365-2133.2007.08160.x

[21] Modaghegh S, Salehian B, Tavassoli M, Djamshidi A, Rezai AS. Use of phenytoin in healing of war and non-war wounds. A pilot study of 25 cases. Int J Dermatol. 1989;28(5):347–50. 2666326 10.1111/j.1365-4362.1989.tb01363.x

[22] Kilkenny C, Browne W, Cuthill IC, Emerson M, Altman DG. Animal research: reporting in vivo experiments: the ARRIVE guidelines. Br J Pharmacol. 2010;160(7):1577–9. 20649561 10.1111/j.1476-5381.2010.00872.x PMC2936830

[23] Patsalos PN, Lascelles PT. Metabolic interactions of phenytoin in the rat: Effect of coadministration with the anticonvulsant drugs sodium valproate, sulthiame, ethosuximide or phenobarbital. Gen Pharmacology: Vascular Syst. 1984;15(1):7–12. 10.1016/0306-3623(84)90072-7 6141984

[24] Taheri Mirghaed M, Ghasemian SO, Mousavi Nasab SF, Rahimi K. Effects of fish oil on ethanol-induced gastric ulcer in rats: inflammatory responses and oxidative stress. Annals Med Surg. 2024;86(2). 10.1097/MS9.0000000000001550 PMC10849447 38333309

[25] Rahimi K, Ezzati Givi M, Rezaie A, Hekmatmanesh M, Shaker Ardakani Y. The protective effects of Gamma-linolenic acid against indomethacin-induced gastric ulcer in rats. Br J Nutr. 2024;131(11):1844–51. 38443203 10.1017/S0007114524000382

[26] Soltani M, Ghotbeddin Z, Rahimi K, Tabandeh MR. Effects of Alpha-pinene on oxidative stress and inflammatory response in acute gastric ulcers in rats. Iran Veterinary J. 2024;20(3):101–13.

[27] Das SK, Vasudevan DM. Alcohol-induced oxidative stress. Life Sci. 2007;81(3):177–87. 17570440 10.1016/j.lfs.2007.05.005

[28] Kemmerly T, Kaunitz JD. Gastroduodenal mucosal defense. Curr Opin Gastroenterol. 2014;30(6):583–8. 25229259 10.1097/MOG.0000000000000124 PMC4238382

[29] Minatel IO, Francisqueti FV, Corrêa CR, Lima GPP. Antioxidant activity of γ-oryzanol: a complex network of interactions. Int J Mol Sci. 2016;17(8):1107. 27517904 10.3390/ijms17081107 PMC5000585

[30] Liu T, Zhang L, Joo D, Sun S-C. NF-κB signaling in inflammation. Signal Transduct Target therapy. 2017;2(1):1–9. 10.1038/sigtrans.2017.23 PMC5661633 29158945

[31] Napetschnig J, Wu H. Molecular basis of NF-κB signaling. Annual Rev Biophys. 2013;42(1):443–68. 23495970 10.1146/annurev-biophys-083012-130338 PMC3678348

[32] Hoesel B, Schmid JA. The complexity of NF-κB signaling in inflammation and cancer. Mol Cancer. 2013;12(1):86. 23915189 10.1186/1476-4598-12-86 PMC3750319

[33] Israël A. The IKK complex, a central regulator of NF-κB activation. Cold Spring Harb Perspect Biol. 2010;2(3):a000158. 20300203 10.1101/cshperspect.a000158 PMC2829958

[34] Winston JT, Strack P, Beer-Romero P, Chu CY, Elledge SJ, Harper JW. The SCFbeta-TRCP-ubiquitin ligase complex associates specifically with phosphorylated destruction motifs in IkappaBalpha and beta-catenin and stimulates IkappaBalpha ubiquitination in vitro. Genes Dev. 1999;13(3):270–83. 9990852 10.1101/gad.13.3.270 PMC316433

[35] Brown K, Gerstberger S, Carlson L, Franzoso G, Siebenlist U. Control of I kappa B-alpha proteolysis by site-specific, signal-induced phosphorylation. Sci (New York NY). 1995;267(5203):1485–8. 10.1126/science.7878466 7878466

[36] Oeckinghaus A, Hayden MS, Ghosh S. Crosstalk in NF-κB signaling pathways. Nat Immunol. 2011;12(8):695–708. 21772278 10.1038/ni.2065

[37] Sun S-C. The non-canonical NF-κB pathway in immunity and inflammation. Nat Rev Immunol. 2017;17(9):545–58. 28580957 10.1038/nri.2017.52 PMC5753586

[38] Maruyama T, Fukushima H, Nakao K, Shin M, Yasuda H, Weih F, Doi T, Aoki K, Alles N, Ohya K, et al. Processing of the NF-kappa B2 precursor p100 to p52 is critical for RANKL-induced osteoclast differentiation. J bone mineral research: official J Am Soc Bone Mineral Res. 2010;25(5):1058–67. 10.1359/jbmr.091032 19874202

[39] Senftleben U, Cao Y, Xiao G, Greten FR, Krähn G, Bonizzi G, Chen Y, Hu Y, Fong A, Sun SC, et al. Activation by IKKalpha of a second, evolutionary conserved, NF-kappa B signaling pathway. Sci (New York NY). 2001;293(5534):1495–9. 10.1126/science.1062677 11520989

